# Thanks to all those who reviewed for *Journal of Negative Results in BioMedicine* in 2015

**DOI:** 10.1186/s12952-016-0046-z

**Published:** 2016-02-17

**Authors:** Bjorn R. Olsen, Dirk Mielenz

**Affiliations:** Department of Cell Biology, Harvard Medical School, Boston, MA USA; Division of Molecular Immunology, Department of Internal Medicine 3, University of Erlangen-Nuremberg, Nikolaus Fiebiger Centre, Erlangen, Germany

## Abstract

A peer-reviewed journal would not survive without the generous time and insightful comments of the reviewers, whose efforts often go unrecognized. Although final decisions are always editorial, they are greatly facilitated by the deeper technical knowledge, scientific insights, understanding of social consequences, and passion that reviewers bring to our deliberations. For these reasons, the Editor-in-Chief, Managing Editor and staff of *Journal of Negative Results in BioMedicine* warmly thank the 51 reviewers whose comments helped to shape the journal, for their invaluable assistance with review of manuscripts in Volume 14 (2015).

**R Abu Bakar**

Malaysia

**Thawatchai Akaraviputh**

Thailand

**Alessandro Alessandrini**

USA

**Jessica S Ancker**

USA

**Mani Arsalan**

Germany

**Aline Bozec**

Germany

**Simon Brill**

UK

**Eugene Chang**

USA

**Hui Chen**

Australia

**Af Cicero**

Italy

**Abdullah Kursat Cingu**

Turkey

**Abdullah Cingü**

Turkey

**Christopher Earley**

USA

**Bjorn Ebdrup**

Denmark

**Jorg Ellinger**

Germany

**Gabor Erdoes**

Switzerland

**Sylvia Fitting**

USA

**V M Freitas**

Brazil

**Christian Gentili**

Switzerland

**Bettina Groetsch**

Germany

**Thomas Harrer**

Germany

**Cheryl Hubley-Kozey**

Canada

**Dirk Hubmacher**

USA

**Sabir Hussain**

Pakistan

**Klaus Jung**

Germany

**Maria Kosmidou**

Greece

**Gerhard Kroenke**

Germany

**Christopher Kushmerick**

Brazil

**Klea Lamnissou**

Greece

**Stefanie Lang**

Germany

**Alejandro Martinez-Fernandez**

Spain

**Daniel Medeiros**

Brazil

**Dirk Mielenz**

Germany

**S Miyata**

Japan

**Pretal Muldoon**

USA

**Bjorn Olsen**

USA

**Caroline Owen**

USA

**D Papazoglou**

Greece

**Manuela Plate**

UK

**Iryna Prots**

Germany

**Juan Carlos Rodriguez-Manzaneque**

Spain

**B Schlehofer**

Germany

**Frederico Soriani**

Brazil

**Athina Tatsioni**

Greece

**Tarnia Taverner**

Canada

**Maria Thomson**

USA

**Ulysses Torres**

Brazil

**Angelo Troina**

Italy

**Dominique Vautiera**

France

**Ye Wang**

China

**Janie Wilson**

Canada

